# Prevalence and risk factors of active hepatitis C infection among at-risk migrant populations in Madrid, Spain, 2019 to 2023

**DOI:** 10.2807/1560-7917.ES.2025.30.29.2500150

**Published:** 2025-07-24

**Authors:** Pablo Ryan, Jorge Valencia, Felipe Pérez-García, Marta Quero-Delgado, Guillermo Cuevas, Samuel Manzano, Samuel Estévez, Isidoro Martínez, Daniel Sepúlveda-Crespo, Salvador Resino

**Affiliations:** 1 Hospital Universitario Infanta Leonor, Madrid, Spain; 2 Universidad Complutense de Madrid (UCM), Madrid, Spain; 3 Instituto de Investigación Sanitaria Gregorio Marañón (IiSGM), Madrid, Spain; 4 Centro de Investigación Biomédica en Red en Enfermedades Infecciosas (CIBERINFEC), Instituto de Salud Carlos III, Madrid, Spain; 5 Unidad de Reducción de Daños ‘SMASD’, Madrid, Spain; 6 Servicio de Microbiología Clínica, Hospital Universitario Príncipe de Asturias, Madrid, Spain; 7 Universidad de Alcalá, Facultad de Medicina, Departamento de Biomedicina y Biotecnología, Madrid, Spain; 8 Unidad de Infección Viral e Inmunidad, Centro Nacional de Microbiología, Instituto de Salud Carlos III, Majadahonda, Madrid, Spain

**Keywords:** Hepatitis C, mobile screening unit, migrant populations, risk factors, injection drug use, Spain

## Abstract

**BACKGROUND:**

Hepatitis C virus (HCV) microelimination among at-risk migrants supports global elimination goals.

**AIM:**

To evaluate risk factors, prevalence and trends of active HCV infection among at-risk migrants screened for HCV in Madrid from 2019–23.

**METHODS:**

At-risk migrants (born outside Spain, living in country < 10 years regardless of legal status), were screened for HCV via mobile units with rapid antibody testing, and confirmed by RNA testing. Recruitment of this convenience sample focused on migrant centres, shelters, harm reduction centres and social service sites. Primary outcome was active HCV prevalence. Risk factors analysed included origin, alcohol use, no stable income, drug use and sexual behaviour. Data were analysed using general linear models with negative binomial distribution and p values adjusted for multiple comparisons (q values).

**RESULTS:**

Among 2,288 migrants, 6.5% (149/2,288) had anti-HCV antibodies, 47.0% (70/149) of whom tested positive for HCV-RNA; 81.4% (57/70) began antiviral therapy. Overall prevalence of active HCV infection was 3.1% (70/2,288). Injection drug use (non-active vs never used (aIRR: 7.3; 95% CI: 2.7–12.7) and active (aIRR: 14.7; 95% CI: 6.7–32.1)), European origin (vs non-European; aIRR: 5.8; 95% CI:  2.7–12.7) and alcohol misuse (vs no misuse; aIRR: 1.8; 95% CI: 1.1–2.9) were main risk factors. Prevalence showed no significant change during 2019–23 in the overall population and across risk groups.

**CONCLUSION:**

At-risk migrants screened in Madrid had a high prevalence of active HCV infection. This is higher than reported estimates for the general Spanish population and supports the need to enhance targeted HCV prevention, screening and treatment strategies among migrant populations.

**Key public health message**

**What did you want to address in this study and why?**

Untreated hepatitis C virus (HCV) infection can lead to severe liver disease, including cirrhosis and cancer. We aimed to assess the prevalence and risk factors of active HCV infection among at-risk migrants (individuals born outside Spain living in the country for < 10 years regardless of legal status) in Madrid, from 2019 to 2023. By understanding the prevalence and identifying key risk factors, we sought to guide targeted interventions and contribute to global HCV elimination.

**What have we learnt from this study?**

Our study revealed that the key risk factors for active HCV infection included injection drug use (IDU), European origin and alcohol misuse. The prevalence of active HCV infection among at-risk migrants in Madrid was 3.1%, compared to 0.22% in the general population. These findings highlight a remarkable health disparity affecting this vulnerable population. Despite increased HCV screening and treatment efforts, the prevalence remained stable over the study period.

**What are the implications of your findings for public health?**

Our findings highlight the need for continued efforts to prevent and treat HCV infection among at-risk migrants in Madrid. Targeted interventions should focus on key groups with high-risk behaviours, particularly those engaging in IDU and alcohol misuse. Additionally, expanding access to HCV screening and treatment, especially for marginalised populations, who often face barriers such as stigma, unstable housing or limited healthcare access, to achieve HCV microelimination.

## Introduction

Untreated hepatitis C virus (HCV) infection progresses to chronic hepatitis C in ca 70% of cases, potentially causing severe liver damage, including cirrhosis, liver failure, and cancer [1]. Direct-acting antivirals (DAAs), with over a 95% success rate and minimal side effects [2], have revolutionised HCV treatment, become the standard of care and contributed to a decline in new cases [3]. Despite this, global access to treatment is limited by cost, insurance coverage, geographical barriers and delayed diagnosis [4].

The World Health Organization (WHO) aims to reduce the global prevalence of HCV by 2030 through expanded screening, risk reduction and universal treatment access [2]. In 2022, an estimated 50 million people (0.7% of the global population) were living with chronic hepatitis C [5], with only 36% aware of their infection and 20% treated [5]. In Spain, the prevalence of HCV declined from 1.2% in 2013 [6] to 0.22% in 2017–18 [7], placing the country on track to meet the WHO’s targets [3]. This progress can be attributed largely to a national strategy launched in 2015 and to unrestricted access to DAAs since 2017 [8]. Targeted microelimination strategies in high-risk groups [9,10], including marginalised populations [11,12], have further accelerated progress. However, undiagnosed infections, particularly in migrants, may hinder elimination efforts.

Migrants comprise an estimated 14% of all hepatitis C cases in the European Union/European Economic Area (EU/EEA) [13], disproportionately affecting those originating from countries or regions outside the EU/EEA with moderate (≥ 2%) or high (≥ 5%) HCV prevalence [14]. Social marginalisation and vulnerability are common among the migrant population, with high rates of poverty, people who inject drugs (PWID), people who use drugs (PWUD), people living with human immunodeficiency virus (HIV), sex workers, homeless individuals and prisoners [15]. These marginalised migrant groups are particularly vulnerable and experience varying HCV prevalence rates. Notably, studies indicate that injection drug use (IDU), a known major risk factor for hepatitis C [12,16], is more common among certain migrant subgroups [17,18].

In Spain, where ca 8.9 million migrants reside (representing 18.3% of the total population as at April 2023) [19] and national HCV prevalence is low, migrants account for a notable proportion (12%) of both chronic and new/recurrent HCV infections [13]. This elevated likelihood of infection is multifactorial, encompassing a combination of factors such as origin from endemic regions [15], limited HCV awareness and prevention knowledge [20], restricted healthcare access leading to delayed diagnosis and poor linkage to care [21,22], overcrowded living conditions [23], adverse experiences during migration or detention [24], and engagement in high-risk behaviours, with IDU being the primary transmission route [25]. While sexual transmission is generally uncommon, it occurs more frequently in other contexts, such as among men who have sex with men engaging in condomless anal intercourse, with multiple sexual partners, or those involved in chemsex [26,27]. Improving the hepatitis C care cascade for migrants is crucial for protecting individual and public health, promoting equitable healthcare access, reducing long-term economic burdens, and preventing disease transmission and outbreaks [28].

This study aimed to evaluate risk factors, prevalence and temporal trends of active HCV infection from 2019 to 2023 among at-risk migrants screened for HCV in Madrid, Spain.

## Methods

### Study design and population

This cross-sectional study was conducted in Madrid, Spain, from February 2019 to April 2023, targeting at-risk migrant populations. For this study, at-risk migrants were defined as individuals born outside Spain who had relocated to the country—regardless of legal status—and had been living in Spain for less than 10 years. Inclusion criteria required the presence of at least one of the following clearly defined psychosocial vulnerabilities that may increase susceptibility to HCV acquisition: homelessness or unstable housing situation, current or past injection drug use, problematic alcohol use, lack of regular income, limited access to healthcare services and origin from a country with moderate (≥ 2%) or high (≥ 5%) HCV prevalence. These psychosocial vulnerabilities reflect a broader context of structural and social determinants—including socioeconomic marginalisation, legal and language barriers, and limited access to healthcare—that increase the risk of HCV through both direct exposure and reduced access to diagnosis and care. 

Eligible participants were adults (≥ 18 years) who were recruited consecutively through targeted outreach activities. These activities were conducted at screening sites located in geographic ‘hotspots’ within Madrid. For this study, a ‘hotspot’ was defined as a specific geographic area or venue known to have high concentrations of, or be frequently attended by, at-risk migrants. These included sites such as homeless shelters, harm reduction centres, addiction treatment facilities, social service institutions and specific neighbourhoods identified by local health authorities and community organisations as areas with limited healthcare access and elevated risk of HCV transmission.

### Mobile hepatitis C virus screening and support services

A mobile unit, accompanied by a support vehicle, was used for HCV screening of at-risk migrants, operating on a pre-established schedule [11,12]. Outreach efforts were conducted in collaboration with community organisations to encourage participation. While no financial incentives were provided, participants received health education designed to facilitate access to healthcare services. During the intervention, a peer navigator/educator collected epidemiological data. Concurrently, a nurse performed point-of-care HCV screening, which included the collection of a capillary blood sample. Following screening, participants were provided with essential harm-reduction materials to prevent HCV acquisition. For individuals diagnosed with HCV, a structured follow-up protocol was implemented, including appointment reminders and peer navigators, to improve retention in care.

Most screening and data collection procedures were straightforward, focusing on essential sociodemographic information to minimise task complexity and language dependency. When language barriers arose, sociocultural mediators or interpreters were used. This support was provided by participants' peers who possessed broader language skills and Spanish proficiency, as well as by staff from collaborating institutions fluent in various participants' native languages.

It is important to note that while a larger global project, of which this study was part, incorporated screening for other blood-borne viruses at various stages, this specific analysis concentrated solely on HCV screening and outcomes among the at-risk migrant population. Screening for other blood-borne viruses, such as hepatitis B virus (HBV) and HIV, was outside the scope of this particular report.

### Screening for active hepatitis C virus infection

Capillary blood samples were tested for anti-HCV antibodies using the OraQuick HCV Rapid Antibody Test (OraSure Technologies). Participants who tested positive for anti-HCV antibodies subsequently underwent confirmatory HCV ribonucleic acid (RNA) testing using the Xpert HCV Viral Load Fingerstick assay (Cepheid) to determine active infection [11,12].

A systematic assessment of HCV reinfection was not conducted for this study. To ensure accurate prevalence estimates and avoid duplication in the analysis, participants with multiple HCV tests within the study period were included only once per calendar year. In instances where a participant underwent more than one test in the same year, only the result of the first test was considered. As a result, potential reinfections occurring within the same calendar year were not captured, which may have resulted in an underestimation of the total number of infections.

### Linkage to care

Following a positive HCV RNA test result, participants—all of whom provided at least one form of contact information— were informed of their diagnosis, counselled on the importance of treatment, and referred to a designated hospital or specialised healthcare facility for further evaluation and treatment initiation. A peer navigator facilitated this referral process by scheduling appointments and providing support to ensure continuity of care. Follow-up calls and in-person reminders were used to improve retention in care and treatment adherence. Clinical follow-up included fibrosis assessment via elastography and evaluation for complications such as cirrhosis and hepatocellular carcinoma.

### Data collection

During the visit at the mobile unit, study participants underwent simultaneous interviews and HCV screening, followed by the provision of crucial harm reduction information to prevent HCV acquisition. A comprehensive set of sociodemographic and epidemiological data was collected via confidential, 10-min-long electronic surveys administered on tablets within a private area of the mobile unit. Data were collected using REDCap (Research Electronic Data Capture) software, as previously described [11,12]. Cultural mediators or interpreters were available to assist participants as needed, ensuring comprehension and accurate responses.

### Risk factors

The risk factors (x) self-reported for this study were as follows: (i) sociodemographic variables: age, sex, origin and calendar year of HCV screening; (ii) social factors: homelessness (defined as lacking stable, regular housing or residing in temporary shelters or public spaces in the previous 6 months; yes/no) and undocumented residency status (defined as residing in Spain without the corresponding authorisation; yes/no); (iii) economic factors: lack of stable income (defined as having no regular or stable financial earnings or support at the time of the survey); (iv) substance abuse variables in the last year: alcohol misuse (defined as self-reported consumption of > 50 g/day; yes/no), benzodiazepine use (defined as self-reported consumption yes/no); (v) PWUD status: use of cocaine, heroin or marijuana (yes/no); (vi) PWID status in the last year: history of IDU (never, non-active or active) and opioid substitution therapy (OST; yes/no); and (vii) sexual behaviour variables: history of sexual activity in the last year (never, condom use and no condom use).

### Outcome variable

The outcome variable (y) of this study was active HCV infection, defined as detectable HCV-RNA among participants who tested positive for anti-HCV antibodies.

### Statistical analysis

We used Stata 17 (StataCorp LLC) for statistical analyses and GraphPad Prism 9 (GraphPad Software, Inc.) for generating figures. P values < 0.05 (two-tailed) were considered statistically significant. Differences between study groups were assessed using the Mann-Whitney U test for continuous variables and either the chi-square or Fisher’s exact test for categorical variables, as appropriate.

The prevalence of active HCV infection was calculated by dividing the number of participants with a positive HCV RNA test by the total number of participants who underwent HCV antibody testing.

General linear models (GLM) with a negative binomial distribution and a log link were used to estimate prevalence ratios (PRs) —reported as incidence rate ratios (IRRs)— for active HCV infection over time (2019–23) and to assess risk factors. This approach allows for direct PR estimation from cross-sectional data and accommodates potential overdispersion in the count of positive cases within defined strata. Standard errors were calculated using bootstrap replications (1,000 times) to ensure the robustness of the confidence intervals, particularly given the complex nature of the study population. All independent variables included in the multivariate GLM had less than 5% missing data. Besides, all risk factors were included in the multivariate regression model, except those with high collinearity, as defined by a Spearman correlation coefficient > 0.5 or variance inflation factor (VIF) > 5.

These GLMs provide adjusted IRRs (aIRRs), 95% confidence intervals (95% CI), and p values. Stata's *pwcompare* command calculated pairwise comparisons between identified risk factors. Because of the extensive number of statistical comparisons in the stratified analysis of risk factors, a multiple testing correction was applied using the Benjamini-Hochberg procedure, adjusting p values to q values.

## Results

### Hepatis C virus screening and diagnostic algorithm

HCV screenings were conducted at 35 diverse locations in Madrid, ranging from small centres with fewer than 10 people examined per screening event to larger facilities, including shelters for homeless individuals, drug rehabilitation centres, addiction treatment centres and social support centres.

**Figure 1:**
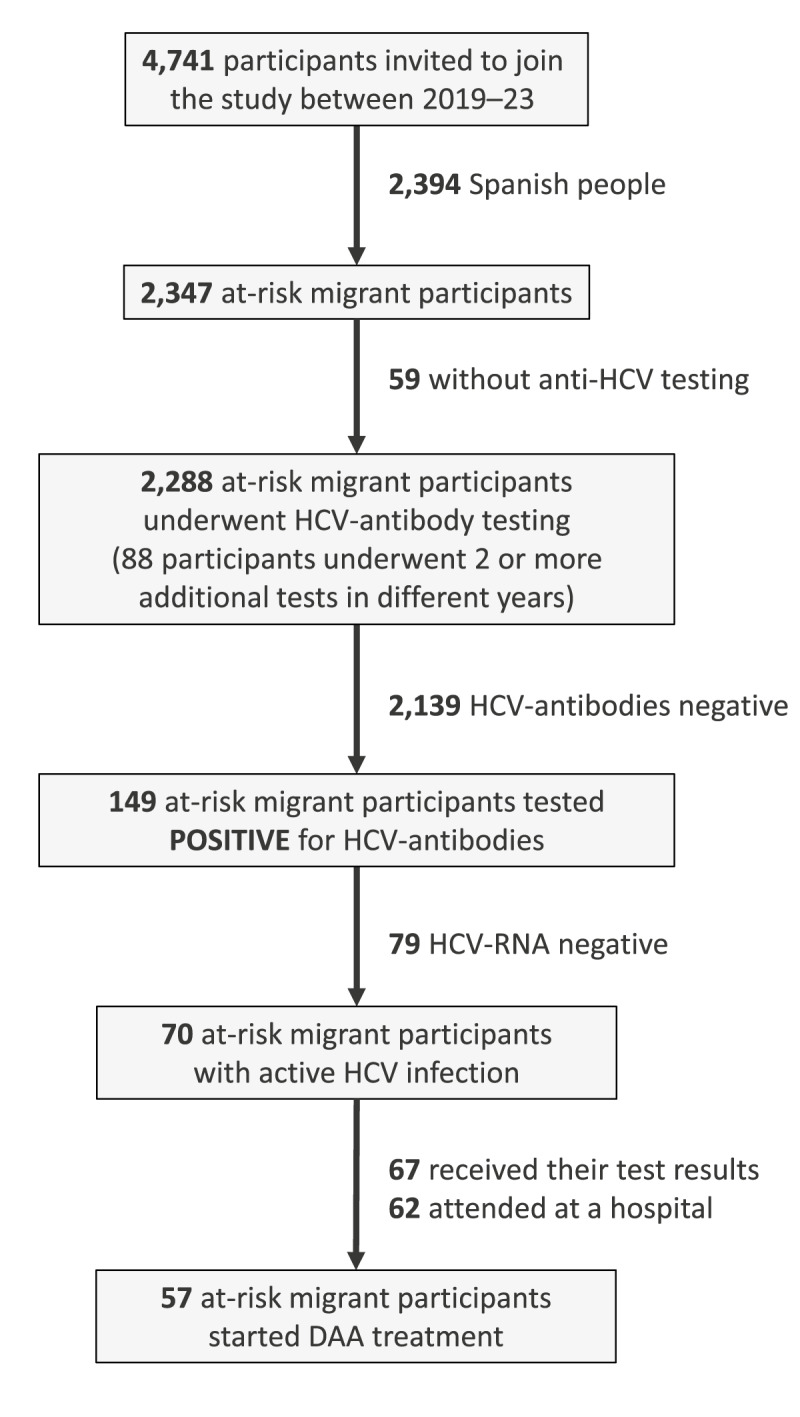
Hepatitis C virus screening and diagnostic algorithm flowchart of the study population, Madrid, Spain, 2019–2023 (n = 2,288 participants)

Figure 1 illustrates the participant flow through the HCV screening and diagnostic algorithm, culminating in linkage to care. A total of 4,741 individuals who visited the mobile screening units were initially invited to participate in the study and screened for eligibility between 2019 and 2023. Of these, 2,394 were identified as Spanish-born individuals and were excluded from further analysis because this study specifically targeted at-risk migrants. This left 2,347 at-risk migrants eligible for inclusion, representing 49.5% (2,347/4,741) of those initially invited. Among this group, 2.5% (59/2,347) of eligible at-risk migrants did not undergo anti-HCV antibody testing because of logistical constraints or refusal. Consequently, the final study population for HCV antibody testing comprised 2,288 at-risk migrant participants (representing 97.5% (2,288/2,347) of eligible at-risk migrants), with the annual distribution as follows: 786 in 2019, 557 in 2020, 331 in 2021, 549 in 2022, and 65 in 2023. It is noted that 3.8% (88/2,288) of these 2,288 participants underwent two or more additional tests in different years. However, for prevalence calculations and to avoid duplication in the primary analysis cohort, each individual was considered only once, based on their first relevant test, or once per year for annual analyses.

DAA: direct-acting antiviral; HCV: hepatitis C virus.

Of those 2,347 screened, 59 individuals were not tested due to logistics and/or refusal.

Of the 2,288 at-risk migrants screened, 6.5% (149/2,288) tested positive for anti-HCV antibodies. All 149 anti-HCV-positive individuals subsequently underwent confirmatory HCV-RNA testing. Among this group, 47.0% (70/149) were confirmed to have active HCV infection (HCV-RNA positive), corresponding to an overall active HCV prevalence of 3.1% (70/2,288) in the screened cohort. Regarding the linkage to care for the 70 individuals with active HCV infection, 95.7% (67/70) received their test results. Among those with an active infection, 88.6% (62/70) attended a follow-up hospital appointment, representing 92.5% (62/67) of those who had received their results. Ultimately, 81.4% (57/70) of individuals with active infection initiated DAA treatment; this constitutes 91.9% (57/62) of those who attended a hospital appointment.

### Participant characteristics

The characteristics of all included participants and those with active HCV infection are summarised in the Table. The median age among at-risk migrants was 38 years (interquartile range (IQR): 30–48). Additionally, 69.1% (n = 1,582) were male, 30.9% (n = 706) were female, and 72.2% (n = 1,652) were experiencing homelessness, 74.4% (n = 1,703) lacked financial income and 23.0% (n = 527) were undocumented at-risk migrants. Regarding substance abuse and PWUD in the last year, 30.7% (n = 703) reported alcohol misuse (> 50 g/day), 17.9% (n = 409) benzodiazepine use, 19.8% (n = 453) cocaine use and 12.8% (n = 292) heroin use. Among PWID, 10.6% (n = 243) reported a history of IDU (of whom 46.5% (n = 113) were active in the last year), and 62.6% (n = 152) of those with a history of IDU received opioid substitution therapy (OST). Regarding sexual behaviour in the last year, 60.2% (n = 1,378) of all at-risk migrants reported being sexually active, and of those, 35.9% (n = 495) who were sexually active did not use condoms.

**Table:** Epidemiological characteristics of at-risk migrants, Madrid, Spain, 2019–2023 (n = 2,288)

Characteristics	All at-risk migrants n = 2,288		Active HCV infection						
			Non-HCV-infected n = 2,218		HCV-infected n = 70			p value	
	n	%	n	%	n	%		n	%
Male	1,582	69.1	1,520	68.5	62	88.6		**< 0.001**	
Female	706	30.9	698	31.5	8	11.4			
Age, median (IQR)	38 (30–48)		38 (29–48)		39 (34–47)			0.356	
Age > 40 years	970	42.4	944	42.6	26	37.1		0.413	
**Social situation**									
Homelessness	1,652	72.2	1,601	72.2	51	72.9		0.637	
Lack of stable income	1,703	74.4	1,647	74.2	56	80.0		0.150	
Undocumented status	527	23.0	514	23.2	13	18.6		0.408	
**Substance abuse (last year)**									
Alcohol misuse (> 50 g/day)	703	30.7	667	30.1	36		51.4	**< 0.001**	
Benzodiazepine	409	17.9	370	16.7	39		55.7	**< 0.001**	
**PWUD (last year)**									
Cocaine	453	19.8	408	18.4	45		64.3	**< 0.001**	
Heroin	292	12.8	244	11.0	48		68.6	**< 0.001**	
Marijuana	227	9.9	222	10.0	5		7.1	0.430	
**PWID (last year)**									
History of IDU	243	10.6	190	8.6	53		75.7	**< 0.001**	
Active^a^	113	46.5	73	38.4	40		75.5	**< 0.001**	
Opioid substitution therapy^a^	152	62.6	120	63.2	32		60.4	0.715	
**Sexual behaviour (last year)**									
Sexual intercourse	1,378	60.2	1,335	60.2	43		61.4	0.581	
No condom use^a^	495	35.9	486	36.4	9		20.9	**0.037**	

HCV: hepatitis C virus; IDU: injection drug use; IQR: interquartile range; PWID: people who inject drugs; PWUD: people who use drugs.

^a^ Percentages for these variables are calculated within the relevant subset of participants.

For statistics, values are expressed as numbers (percentage) and median (interquartile range). Differences between study groups were assessed using the Mann-Whitney U test for continuous variables and either the chi-square or Fisher’s exact test for categorical variables, as appropriate. Significant values (p  < 0.05) are indicated in bold text.

### Region of origin and active HCV infection

**Figure 2:**
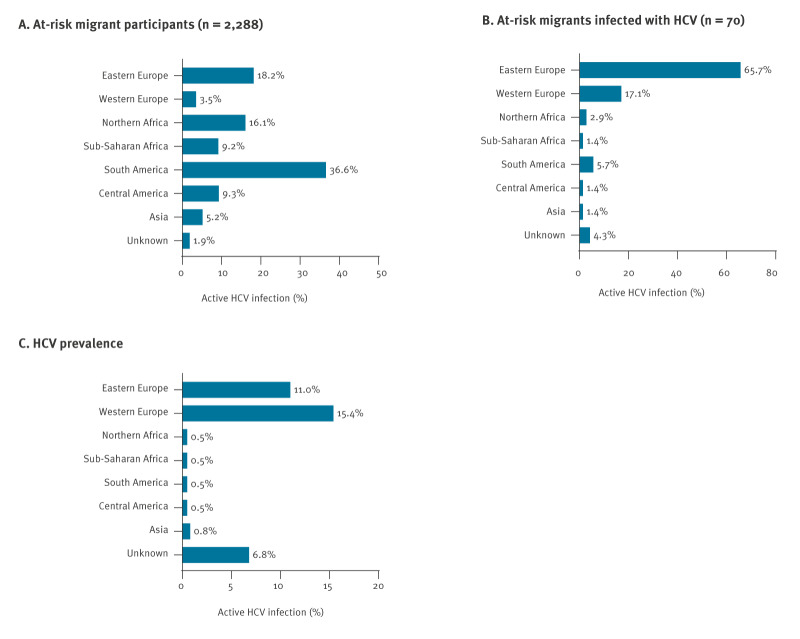
Regions of origin of all at-risk migrants (A) and those with active HCV infection (B), and prevalence (C), Madrid, Spain, 2019–2023 (n = 2,288)

A summary of the countries of origin for the studied population, stratified by region, listing all individual countries included within each region, is presented in Supplementary Table S1. Over one-third of the at-risk migrants originated from South America (36.6%; 837/2,288). Other prominent regions of origin included Eastern Europe (18.2%; 417/2,288) and North Africa (16.1%; 369/2,288) (Figure 2A). Migrants from Western Europe were the least numerous, accounting for 3.4% (78/2,288).

HCV: hepatitis C virus.

During the study period (2019–23), the overall prevalence of active HCV infection was 3.1% (70/2,288; 95% CI: 2.4–3.8). However, active HCV infection was predominantly observed among at-risk migrants from Eastern Europe and Western Europe. Specifically, individuals from Eastern Europe accounted for 65.7% (46/70) of all active HCV cases, and those from Western Europe accounted for 17.1% (12/70) (Figure 2B). These were the only two regions of origin presenting a prevalence of active HCV infection much higher than 1%, at 11.0% (46/417) for Eastern Europe and 15.4% (12/78) for Western Europe, respectively (Figure 2C).

### Risk factors and active hepatitis C virus infection prevalence

**Figure 3:**
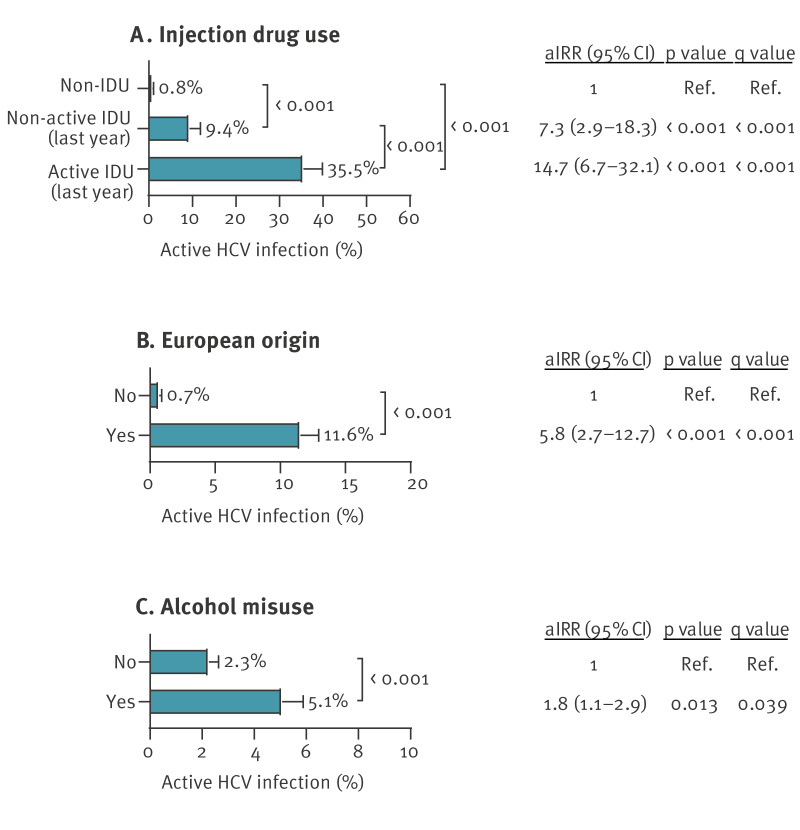
Risk factors for active hepatitis C virus infection in at-risk migrants, Madrid, Spain, 2019–2023 (n = 2,288)

In the multivariate regression analysis, high collinearity was identified for both the calendar year in which HCV screening was conducted (VIF = 14.1) and OST (r = 0.76 with IDU). These two variables were therefore excluded. The final multivariate GLM identified three significant risk factors associated with the active HCV infection. Supplementary Table S2 provides detailed statistical results of the model, including all variables analysed. The significant factors were: IDU (non-active (aIRR = 7.3; 95% CI: 2.9–18.3) and active (aIRR = 14.7; 95% CI: 6.7–32.1)); Figure 3A), European origin (aIRR = 5.8; 95% CI: 2.7–12.7; Figure 3B), and alcohol misuse (aIRR = 1.8; 95% CI: 1.1–2.9; Figure 3C). When stratifying the study population by the three significant risk factors, at-risk migrants of European origin (cf.d with non-European origin) had a higher proportion of non-active IDU (11.9% (59/496) vs 4.1% (73/1,792); p < 0.001), active IDU (17.3% (86/496) vs 1.5% (27/1,792); p < 0.001) and alcohol misuse (44.6% (221/496) vs 26.9% (482/1,792); p < 0.001).

aIRR: adjusted incidence rate ratio; CI: confidence intervals; HCV: hepatitis C virus; IDU: injection drug use; Ref.: reference.

For the statistical analysis, data were calculated using general linear models with a negative binomial distribution adjusted by patient characteristics and bootstrap repetitions (1,000). P values were corrected by the Benjamini-Hochberg procedure (q value).

**Figure 4:**
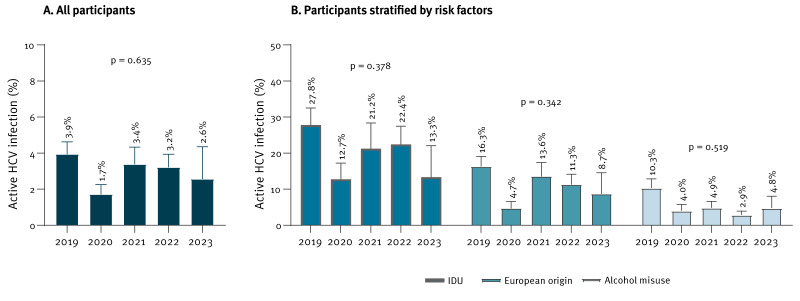
Estimating the prevalence of active hepatitis C virus infection and temporal trends among at-risk migrants by calendar year, Madrid, Spain, 2019–2023 (n = 2,288)

The prevalence of active HCV infection during the study period (2019–23) showed no statistically significant trend over time, neither in the overall population nor when stratified by the three main risk factors (Figure 4).

HCV: hepatitis C virus; IDU: injection drug use.

For the statistical analysis, data were calculated using general linear models with negative binomial distribution and bootstrap repetitions (1,000).

## Discussion

This study of at-risk migrants screened in Madrid (2019–23) reveals two principal findings with important public health implications for HCV elimination, though the results should be interpreted with caution given the limited sample size and specific setting. Firstly, we identified a high prevalence of active HCV infection (3.1%), ca 14-fold higher than the contemporaneous estimate of 0.22% for the general Spanish population [7]. The primary drivers of this disproportionate burden were a history of IDU, European origin and alcohol misuse. Secondly, contrary to the downward trends observed in other local vulnerable populations [11,12], the prevalence of active HCV infection within this specific at-risk migrant cohort remained stable throughout the study period, underscoring persistent transmission and the necessity for tailored public health interventions.

Despite the challenges, linkage to care within our diagnosed cohort was encouraging, with 81.4% initiating DAA treatment. Nevertheless, 18.6% (13/70) of those who did not initiate treatment highlight persistent gaps, even post-diagnosis. This loss to follow-up is a known concern in migrant health, often attributed to factors such as mobility, complex healthcare navigation, and diverse barriers to continued engagement [20].

Our study revealed a substantial burden of chronic HCV, with 47.0% of anti-HCV positive migrants having an active infection. While DAA treatment is highly effective, and 81.4% of our diagnosed cohort began therapy, the overall prevalence of active HCV infection among this at-risk migrant group did not decrease over the study period. This suggests that ongoing transmission or persistent barriers to treatment access and completion are counteracting the benefits of therapy. To effectively reduce HCV prevalence and meet elimination goals in this vulnerable population, it's crucial to investigate treatment adherence, completion and reinfection rates, specifically within migrant cohorts.

The identified risk factors align with, yet also add nuance to, the existing European literature [29]. Injection drug use was the most potent predictor (aIRR for active IDU: 14.7), with an active HCV prevalence of 35.4% among at-risk migrants having a history of IDU vs 0.8% among those without. This magnitude is consistent with IDU being the primary driver of HCV transmission globally [30,31]. While a higher proportion of IDU within this at-risk migrant cohort compared with the general population contributes remarkably to the overall higher HCV prevalence, our multivariate analysis identified European origin as an independent risk factor (aIRR: 5.8), even after adjusting for IDU. This suggests that factors beyond simply a higher prevalence of known risk behaviours are at play for certain migrant subgroups. Our stratified analysis further indicated that at-risk migrants of European origin within our cohort exhibited significantly higher frequencies of both non-active IDU, active IDU and alcohol misuse compared with non-European migrants. This suggests that the elevated HCV risk among European migrants in this ‘at-risk’ sample may be substantially mediated by a higher prevalence of these known behavioural risk factors, potentially compounded by factors such as higher HCV endemicity in some countries of origin [32-34] or pre-migration exposures and possibly different patterns of healthcare engagement or specific transmission networks not fully captured by broad risk categories. Alcohol misuse (aIRR: 1.8) as a third factor reinforces concerns about its impact on HCV acquisition and its known role in impeding access to care [29,35]. This challenge can be critical for migrant populations even with ostensibly universal DAA access [36].

The observed stability in active HCV prevalence among this at-risk migrant group over 5 years is a salient finding, particularly as it contrasts with documented declines in HCV prevalence in the general population [37] and among other marginalised groups in Madrid, such as PWUD and individuals experiencing homelessness not specifically stratified by migrant status [11,12]. This disparity suggests that at-risk migrants face distinct, or more profound, barriers to accessing HCV prevention, diagnosis and treatment services compared with other local vulnerable populations. These may include linguistic, cultural, legal and socioeconomic obstacles, potentially exacerbated during the study period by the coronavirus disease (COVID-19) pandemic's disruption of healthcare services and its disproportionate impact on vulnerable communities [38]. Such stable prevalence necessitates a re-evaluation of current public health strategies, pointing towards an urgent need for culturally adapted, low-threshold interventions specifically designed for at-risk migrant communities. Achieving elimination goals requires addressing populations such as migrants in transit, where tracking outcomes and ensuring linkage to care are notably complex [32,39].

Our overall active HCV prevalence of 3.1% is situated within a varied European landscape of reported rates among migrant populations. For instance, studies in the Netherlands reported rates between 0.5 and 1.2% [40-42], Germany 0.7% [43], France 2.7% [44], and Italy 0.7–1.5% [30,31,45]. Direct comparisons are, however, complicated by substantial heterogeneity in study designs, definitions of ‘migrant’, recruitment strategies (e.g. community-based vs clinic-based), and the specific vulnerabilities of the populations studied. Our study's explicit focus on ‘at-risk’ migrants—defined by recent arrival and specific psychosocial vulnerabilities and recruited from ‘hotspots’ such as shelters and harm reduction centres—characterises a population likely at higher baseline risk than broader migrant samples included in some national estimates. While this targeted approach limits generalisability to the entire migrant population in Madrid, it effectively identifies a high-burden subgroup where interventions are most critically needed. Data on active HCV infection among migrants in Spain remain limited; most estimates rely on seroprevalence [15], which does not distinguish between active and resolved infections. A recent Catalan study reported active HCV rates of 1.4–2.4% [46], which, although from a different regional context, are closer to our findings than the seroprevalence estimates.

A key strength of this study is the systematic use of HCV-RNA testing for all individuals who are anti-HCV positive, providing a robust estimate of active infection prevalence over a multi-year period in a large, well-characterised cohort of at-risk migrants. The identification of stable prevalence, despite national elimination efforts, and the elucidation of specific risk factor contributions within this vulnerable group constitute its main added value. These findings challenge the efficacy of current 'one-size-fits-all' approaches and strongly advocate for the development and implementation of nuanced, community-engaged, and culturally competent public health strategies to effectively address HCV within at-risk migrant populations across Europe. Future research should focus on evaluating such tailored interventions to ensure progress towards HCV elimination targets is equitable and inclusive of these often marginalised communities.

This study has several limitations. Firstly, the convenience sampling approach, targeting high-risk locations (shelters, harm reduction centres, social assistance institutions), may limit generalisability to the broader Madrid migrant population and potentially overrepresent individuals with substance use or other vulnerabilities. Secondly, varying screening locations and populations each year could introduce selection biases when interpreting trends in HCV prevalence. Thirdly, the small sample of HCV-positive cases may have limited statistical power to detect subtle trends. Fourthly, some migrants may have declined participation because of factors such as a lack of incentives, language barriers, cultural norms or concerns about their legal status. Furthermore, potential inaccuracies in self-reported data on account of limited Spanish proficiency are acknowledged; however, the availability of cultural mediators and interpreters helped mitigate this issue. Fifthly, the cross-sectional design hinders the establishment of causal relationships between risk factors and active HCV infection. Sixthly, self-reported behavioural data may be subject to bias. Finally, we did not determine the length of time migrants stayed in Spain, nor were HCV genotypes analysed, which limits a deeper understanding of transmission patterns, country-of-origin influences and potential variations in treatment response.

## Conclusions

Active HCV infection prevalence is high among at-risk migrants in Madrid, defined as individuals born outside Spain living in the country for < 10 years regardless of legal status, far exceeding that of the general Spanish population, with IDU, European origin, and alcohol misuse as key risk factors. This disproportionate burden presents a notable public health challenge. The persistent high prevalence of HCV in this migrant subgroup underscores the urgent need for tailored interventions, as existing strategies may be insufficient. Focused research is crucial to developing effective, personalised approaches for HCV elimination in this vulnerable population.

## Data Availability

The datasets used and analysed during the current study are available from the corresponding author upon reasonable request.

## Supplementary Data

221000 Supplement
