# Supplementary material for: Prevalence and risk factors of active hepatitis C infection among at-risk migrant populations in Madrid, Spain, 2019 to 2023
Source: Euro Surveill. 2025 Jul 24;30(29):2500150. doi: 10.2807/1560-7917.ES.2025.30.29.2500150 (PMC12314475; doi:10.2807/1560-7917.ES.2025.30.29.2500150)
Supplement: Supplement [file 25-00150_RESINO_Supplement.pdf]

Supplementary Materials

This supplementary material is hosted by Eurosurveillance as supporting information alongside the article [Prevalence and risk factors of active hepatitis C infection among at-risk migrant populations in Madrid, Spain, 2019 to 2023], on behalf of the authors, who remain responsible for the accuracy and appropriateness of the content. The same standards for ethics, copyright, attributions and permissions as for the article apply. Supplements are not edited by Eurosurveillance and the journal is not responsible for the maintenance of any links or email addresses provided therein.

Supplementary Table S1. Summary of the countries of origin of the studied at-risk migrant population stratified by regions.

Eastern Europe	No. participants	Anti-HCV(+)	HCV-RNA(+)
Bosnia and Herzegovina	1		
Bulgaria	58	27	12
Croatia	3		
Slovakia	4	2	1
Slovenia	2		
Georgia	31	14	10
Hungary	4		
Latvia	2		
Lithuania	4	2	2
Moldova	9	2	1
Poland	45	5	1
Czech Republic	1		
Republic of Macedonia	1		
Romania	188	19	9
Russia	15	4	3
Serbia	2		
Ukraine	47	15	7
**Total**	**417**	**90**	**46**
**Western Europe**			
Germany	6	2	1
Austria	1		
Finland	1	1	1
France	5		
Greece	7	4	1
Italy	20	5	2
Netherlands	1		
Portugal	28	9	7
United Kingdom	10		
**Total**	**79**	**21**	**12**
**Northern Africa**			
Algeria	44	1	
Egypt	1		
Ethiopia	2	1	1
Libya	5	1	1
Morocco	291	6	
Mauritania	3		
Sahara	8		
Somalia	11		
Sudan	2	1	
Tunisia	2		
**Total**	**369**	**10**	**2**
**Sub-Saharan Africa**			
Angola	4		

Cape Verde	2	1	1
Cameroon	21		
Ivory Coast	15		
Gambia	4		
Ghana	9		
Guinea	22		
Equatorial Guinea	21	2	
Guinea-Bissau	2		
Kenya	1		
Liberia	1		
Mali	28		
Nigeria	33		
Central African Republic	1	1	
Democratic Republic of the Congo	1		
South African Republic	2		
Rwanda	1		
Senegal	35		
Sierra Leone	4	1	
Togo	3		
Uganda	1		
**Total**	**211**	**5**	**1**
**South America**			
Argentina	23		
Bolivia	11		
Brazil	31		
Chile	9	1	1
Colombia	229	2	
Ecuador	86		
Paraguay	8		
Peru	100		
Uruguay	11		
Venezuela	329	6	3
**Total**	**837**	**9**	**4**
**Central America**			
Costa Rica	4		
Cuba	52	1	
Dominica	1		
El Salvador	33		
Guatemala	1		
Haiti	1		
Honduras	34		
Jamaica	2		
Mexico	9	1	1
Nicaragua	13		
Panama	4		
Dominican Republic	59		
**Total**	**213**	**2**	**1**

**Asia**			
Afghanistan	55		
Bangladesh	1	1	
Burma	2		
China	10		
Philippines	4		
Iran	1	1	
Iraq	7		
Israel	1		
Japan	1		
Jordan	2		
Kazakhstan	4		
Mongolia	1		
Nepal	1	1	1
Pakistan	3		
Palestine	10	2	
Singapore	1		
Syria	7		
Sri Lanka	2		
Yemen	5		
**Total**	**118**	**5**	**1**
**Unknown**			
Unknown	44	7	3
**Total**	**44**	**7**	**3**

Supplementary Table S2. Factors associated with the active HCV infection among at-risk migrant people

	aOR (95%CI)	p-value	q-value
**Age > 40 years (yes/no)**	0.8 (0.5 - 1.3)	0.326	0.489
**Sex (male/female)**	1.7 (0.6 - 4.4)	0.317	0.489
**European origin (yes/no)**	5.8 (2.7 - 12.7)	**<0.001**	**<0.001**
**Social situation**			
**Homelessness (yes/no)**	0.8 (0.4 - 1.6)	0.616	0.739
**Lack of financial income (yes/no)**	1.5 (0.7 – 3.3)	0.284	0.489
**Undocumented status (yes/no)**	1.1 (0.6 - 1.9)	0.780	0.851
**Substance abuse (last year)**			
**Alcohol misuse (yes/no)**	1.8 (1.1 - 2.9)	**0.013**	**0.039**
**Benzodiazepine (yes/no)**	1.4 (0.8 - 2.4)	0.284	0.489
**PWID (last year)**			
**Non-IDU (reference)**	1	-	
**Non-active IDU**	7.3 (2.9 - 18.3)	**<0.001**	**<0.001**
**Active IDU**	14.7 (6.7 - 32.1)	**<0.001**	**<0.001**
**Sexual intercourse (last year)**			
**No (reference)**	1	-	
**Condom use**	0.8 (0.5 - 1.3)	0.404	0.539
**No condom use**	1 (0.4 - 2.4)	0.976	0.976

Statistical analysis: Data were calculated using a general linear model with a negative binomial distribution, adjusted by patient characteristics and bootstrap repetitions (1,000). The reference category for dichotomous variables was always the one indicated in second place in brackets. *P*-values were corrected by the Benjamini and Hochberg procedure (*q*-value). Statistically significant differences are shown in bold.

Abbreviations: aOR = adjusted odds ratio; HCV = hepatitis C virus; IDU = injection drug use; PWID = people who inject drugs
